# Acquired Hemophilia Associated with Rheumatoid Arthritis: A Case Report and Review of the Literature

**DOI:** 10.3390/ijms26083628

**Published:** 2025-04-11

**Authors:** Chiara Gioia, Marino Paroli, Valentina Morace, Lucrezia Nardacci, Sara Martina Ruffo, Elisabetta Rossi, Pasquale Pignatelli, Daniele Accapezzato

**Affiliations:** Division of Clinical Immunology, Department of Clinical, Anesthesiologic and Cardiovascular Sciences, Sapienza University of Rome, 00185 Rome, Italy; chiara.gioia@uniroma1.it (C.G.); marino.paroli@uniroma1.it (M.P.); valentina.morace@uniroma1.it (V.M.); lucrezia.nardacci@uniroma1.it (L.N.); saramartina.ruffo@uniroma1.it (S.M.R.); e.rossi@policlinicoumberto1.it (E.R.); pasquale.pignatelli@uniroma1.it (P.P.)

**Keywords:** acquired hemophilia, rheumatoid arthritis, FVIII inhibitors

## Abstract

A 63-year-old woman with rheumatoid arthritis and Hashimoto’s thyroiditis was admitted to the emergency room, because of left leg pain associated with spontaneous subcutaneous hematomas, for 15 days. Their symptoms also occurred after the discontinuation of aspirin, which the patient had taken for a previous case of ocular papillitis. Laboratory tests showed anemia, a normal platelet count, but a prolonged activated partial thromboplastin time (aPTT) ratio; a computerized tomography scan of the left lower limb detected a recent hematoma in the left lateral rectus muscle, and subcutaneous soft tissue edema also involving the knee, without vascular involvement. Coagulation tests were performed showing normal levels of Lupus Anticoagulant, very low-factor FVIII activity (2.2%), normal FIX, FXI, and FXII activity, and the detection of FVIII inhibitors by a Bethesda assay (7.6 U). A diagnosis of acquired hemophilia A (AHA) was made, and hemostatic and immunosuppressive treatment was immediately started (activated prothrombin complex concentrates and methylprednisolone). Malignancies and infections were excluded. An autoantibodies panel confirmed the positivity to rheumatoid factor and anti-cyclic citrullinated peptide antibodies. In treatment, the patient did not present any new bruises, with aPTT normalizing, FVIII increasing, and inhibitors reducing until disappearance. A close follow-up continued every 1–2 week after discharge, with hemostatic treatment discontinuation and methylprednisolone decalage. Underlying autoimmune conditions induced this rare, autoimmune and life-threating disorder.

## 1. Introduction

Acquired hemophilia A (AHA) is a rare bleeding disorder related to autoantibodies, called inhibitors, acting against coagulation factor VIII (FVIII). FVIII inhibitors lead to an increased clearance and neutralization of FVIII. AHA is therefore considered an autoimmune coagulation disorder, due to the development of inhibitory autoantibodies against FVIII in individuals without a particular genetic predisposition or history of bleeding. AHA is different from congenital hemophilia, which is hereditary, resulting from mutations in the FVIII gene and it usually is a permanent condition manifesting in early childhood [1]. AHA prevalence ranges from one to four cases per million individuals with a very low incidence rate (0.2–1.0 per 1 million people per year) but it is characterized by a high mortality rate (5–10%) [2].

Men and women are equally affected, with no prevalence by age and with two peaks of incidence: one associated with pregnancy and another with old age (over 60) [3]. Clinically, AHA presents spontaneous bleeding into the skin, muscles, gastrointestinal tracts etc. with highly variable hemorrhagic phenotypes, ranging from mild to life-threatening bleeds. Subcutaneous hematomas are the most typical clinical manifestation. Joint bleeds, the hallmark of congenital hemophilia, are much less common in AHA [4]. Patients classically present with an isolated prolonged activated partial thromboplastin time (aPTT) due to FVIII activity deficiency. The detection of FVIII inhibitors is usually obtained using the modified Bethesda Nijmegen assay [5]. In about 50% of patients, it is possible to recognize an underlying medical condition, including autoimmune diseases, solid tumors, lymphoproliferative malignancies, and pregnancy [6,7]. Hemostatic treatment is based on bypassing agents, including recombinant activated factor VII, activated prothrombin complex concentrate, or recombinant porcine FVIII in patients with hemorrhages. At the same time, inhibitor eradication can be achieved with immunosuppressive therapy, including the use of glucocorticoids (GCs), cytotoxic agents, and rituximab, or combinations of these. After diagnosis, the 2009 international AHA guidelines recommend starting immunosuppressive therapy immediately with all patients with AHA [1]. Adverse events are recorded in about 50% of patients, even after three to four months. Usually, remission is achieved after a median time of 5 weeks. Due to individual variability, the frequency of monitoring must be very high: weekly for the first six weeks, monthly for the first six months. In the follow-up, the preferred evaluations are of FVIII activity and of the FVIII inhibitor titer compared to aPTT [8].

## 2. Case Description

In November 2024, a 63-year-old Russian woman was admitted to the emergency room, because of pain in her left leg associated with spontaneous subcutaneous hematomas, for 15 days. She had been living in Italy since the age of 20. She was affected by rheumatoid arthritis (RA), Hashimoto’s thyroiditis, arterial hypertension, and dyslipidemia, and was undergoing treatment with a low dose of methylprednisolone (4 mg/die), levothyroxine, angiotensin-converting enzyme (ACE) inhibitor, and statin. She was also taking low-dose aspirin (LDA) for a papillitis in her right eye, which had occurred in 2020. Antiphospholipid (aPL) antibodies had tested negative in all previous detections. After discontinuing LDA, new spontaneous hematomas appeared. She denied that any trauma or violence had occurred.

**Figure 1:**
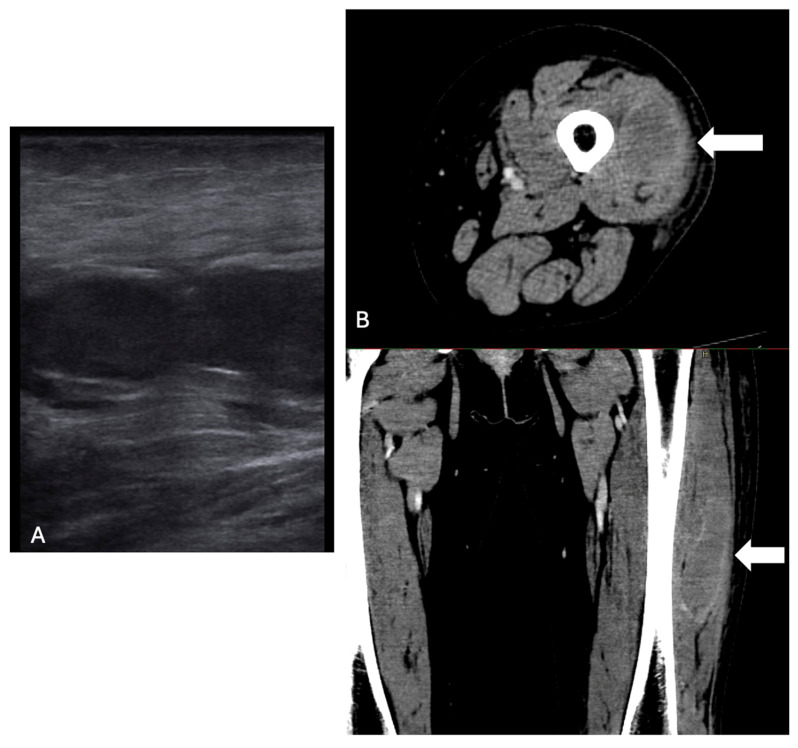
Left lateral rectus muscle hematoma at ultrasound (**A**) and a computerized tomography (**B**) scan (indicated with white arrows).

In the emergency room, her left lower limb was edematous and painful with ecchymosis and swelling in the lateral region and in her left knee. The patient was apyretic and her vital signs were as follows: blood pressure (BP) 120/60 mmHg, heart rate (HR) 74 bpm, oxygen saturation (sO_2_) 98% aa, Glasgow Coma Scale (GCS) 15, and Body Temperature (BT) 36.6 °C. Peripheral pulses of the lower limbs were present. No other region was affected by spontaneous bleeding. Laboratory tests revealed the following: hemoglobin (Hb) 10.6 g/dL, white blood cells (WBC) 12.27 × 10^9^/L, neutrophils (N) 9.35 × 10^9^/L, lymphocytes (L) 1.56 × 10^9^/L, platelets (PLT) 438 × 10^9^/L, lactate dehydrogenase (LDH) 319 U/L, international normalized ratio (INR) 0.92, aPTT ratio 1.84, C-Reactive Protein (CRP) 3.45 mg/dL. An ultrasound and a computerized tomography (CT) scan of the left lower limb were performed, detecting a recent hematoma in the left lateral rectus muscle, subcutaneous edema of the soft tissues, and imbibition also involving the knee, without arterial stenosis, occlusions, and ab extrinseco compression (Figure 1). Compartment syndrome was ruled out.

The patient was then admitted to the Department of Internal Medicine. She was alert, oriented, and she had mild Cushing habitus symptoms (moon face, striae, buffalo hump, and central obesity), for which she was undergoing long-term therapy with low doses of glucocorticoids. Cardiac, thoracic, abdominal, and neurological examinations were negative. At rheumatological examination, only the left knee was tender and swollen with an adjacent hematoma; the typical wrist deformities of bilateral RA were observed. Before and during hospitalization, no episodes of epistaxis, hemoptysis, hemoptysis, hematemesis, melena, gingival, or rectal bleeding were observed. After Medical Department admission, new bruises appeared at the right elbow and hand. The patient presented with normochromic normocytic anemia with a further reduction in Hb, a normal value of PLT, and a confirmed prolonged aPTT. Transferrin saturation was about 10%, folates and vitamin B12 were normal, total and indirect bilirubin were mildly increased with low haptoglobin levels. The results of all laboratory data are reported in Table 1.

Coagulation tests were performed immediately, after prolonged aPTT confirmation. Mixed tests revealed Lupus Anticoagulant (LA) in range, thus indicating a deficiency of coagulation factors. FVIII was severely reduced (2.2%, normal range 58.0–130.0), with normal FIX, FXI, and FXII activity. Both von Willebrand factor (vWF), ristocetin cofactor activity (vWF:RCo), and VWF antigen (vWF:Ag) levels were normal, and antibodies against FVIII were detected with the Bethesda method (7.6 U. Bethesda, normal value < 0.55).

A diagnosis of AHA was made and both hemostatic and immunosuppressive treatments were started, which included activated prothrombin complex concentrates (Feiba^®^) of 80 U/kg every 12 h (6000 U every 12 h) and methylprednisolone 1 mg/Kg/die (80 mg), respectively.

At the same time, a diagnostic iter to recognize an underlying medical condition as a trigger of AHA was carried out. Infections were excluded due to the absence of symptoms and the negativity of the microbiological test. A serum autoantibodies panel was performed: rheumatoid factor (RF) and anti-cyclic citrullinated peptide antibodies (ACPA) were present.

An RA diagnosis was made in 2006: hands, wrists, and knees were the joints involved. The positivity of RF and ACPA were already known. She was treated with non-steroidal anti-inflammatory drugs (NSAIDs), GCs, and methotrexate (MTX). The latter was discontinued for adverse events. The patient’s medical history also revealed the presence of papillitis in the right eye. Anterior optic neuropathy appeared in 2020 after pain and reduced visual acuity. Clinical examination with fundus and brain magnetic resonance imaging (MRI) revealed papillitis, so GCs were administered at a high dosage due to the inflammatory nature of the disease, NSAIDs and MTX were discontinued, and aspirin was started to prevent the ischemic aspect of neuropathy. The patient continued to take aspirin from 2020 until the bruises appeared, in 2024. At the time of hospitalization, RA was in remission, with only longstanding bilateral wrist deformities. Antiphospholipid was permanently negative.

In addition, a total body CT was performed to exclude a neoplastic disease: “A focal pleural thickening is observed (8 mm) at the level of the left lower lobe, in correspondence of the lateral arch of the VIII rib, with progressive contrast enhancement and internal calcifications… some millimetric solid calcific nodules, bilaterally…; hypodense formation (10 mm) at the pancreas head level, probably a Intraductal papillary mucinous neoplasm (IPMN)”. For focal pleural thickening, a Positron Emission Tomography (PET)-CT scan was programmed, and a QuantiFERON TB-Gold test was performed with an indeterminate result on two occasions, probably due to chronic glucocorticoid intake. Abdomen MRI confirmed the presence of cystic formations (approximately 7 mm), localized at the level of the pancreas uncinate process, some with communication with the ductal system, and possible branch–duct BD-IPMN.

Extensive work-up was performed to determine other causes of anemia, including direct and indirect Coombs tests, which were negative. Esophagogastroduodenoscopy and colonoscopy did not reveal any possible causes of bleeding from the gastrointestinal tract. Intravenous iron infusion resulted in a final HB value of 10.7 g/dL.

**Figure 2:**
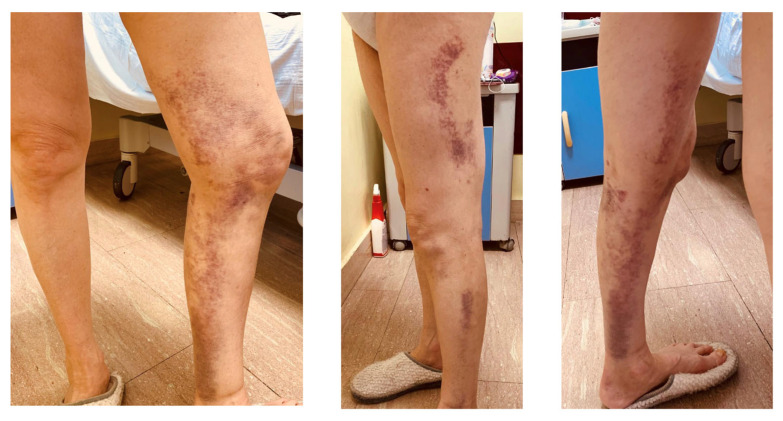
Left limb hematoma tardive evolution.

**Figure 3:**
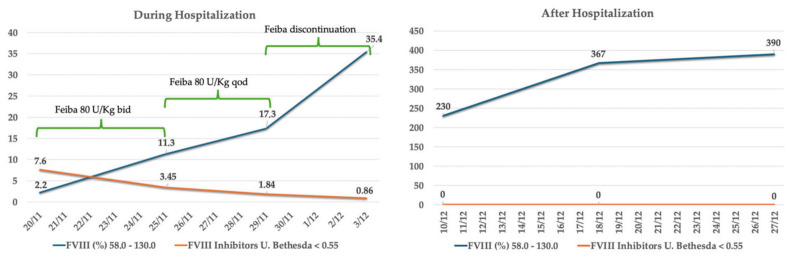
FVIII and FVIII inhibitors during and after hospitalization.

After one-week, Feiba^®^ was administered every other day and after another three days it was discontinued, while FVIII and inhibitor levels were monitored. Methylprednisolone was continued at the same dose. The patient was discharged from hospital with a hematoma in tardive evolution (Figure 2) and the follow-up is ongoing every two weeks at the Hematology Department. Figure 3 shows FVIII and FVIII inhibitor trends during and after hospitalization. She no longer has spontaneous bleeding and the decalage of glucocorticoid therapy has started (prednisone 50 mg).

## 3. Discussion

In this article, we describe a rare case of association between AHA and RA. AHA is a rare autoimmune disease with high morbidity and mortality rates. Unlike congenital hemophilia, AHA is typically a disease of the elderly, with a median age of 64–78 years [9]. Young people are involved when AHA is associated with pregnancy and with autoimmune diseases [10]. Rarely, AHA affects pediatric patients, with severe and occasionally life-threatening hemorrhages due to maternal transplacental transfer of the autoantibody to the newborn [11]. In half of the cases, it is idiopathic whereas in the other 50% of cases another medical condition coexists. Malignancies (solid tumors or lymphoproliferative disorders) are the most common coexisting conditions, followed by autoimmune disorders (especially RA), Hashimoto’s thyroiditis, and pemphigus. Other conditions associated with AHA are infections and some drugs including penicillin and type-I interferon [12,13,14] have also been associated.

Factor VIII acts as a cofactor to factor IXa in the tenase complex, so factor VIII deficiency reduces thrombin generation on the surface of activated platelets. After proteolytic processing, FVIII associates with vWF in the heterodimers of light and heavy chains [15]. Most acquired FVIII inhibitors bind to the A2, A3, or C2 domains. Anti-C2 antibodies interrupt the binding of FVIII to phospholipid and vWF, while antibodies in domains A2 and A3 interfere with FVIII binding to factor X and factor IXa [16]. In acquired hemophilia, autoantibodies are characteristically non-complement fixing, non-precipitating immunoglobulins (Ig) from the IgG family that bind FVIII in a time- and temperature-dependent manner [17]. Factor VIII autoantibodies may be oligoclonal with a mixed composition of IgG subtypes, including IgG1, IgG2, IgG3, and IgG4. Autoantibodies acting against FVIII have also been observed in healthy individuals, in about 19% of cases; the main IgG subtypes are IgG1 and IgG3 which usually bind to non-functional domains of factor VIII, lacking their neutralizing capacity [18]. On the contrary, IgG1 and IgG4 have been reported to be the major components of anti-factor VIII antibodies in patients with AHA [19]. Another difference with congenital hemophilia is the type of inhibitors involved. In congenital hemophilia, most inhibitors (alloantibodies against exogen FVIII) are categorized as “type 1”, resulting in linear inactivation so they can completely neutralize FVIII when present in high concentrations. In contrast, most autoantibodies in AHA are “type 2”, presenting a non-linear, complex inactivation pattern. These bind to FVIII domains in a time- and temperature-dependent manner, following second-order kinetics, significantly reducing FVIII activity [20]. Although some residual FVIII activity could persist, it is not clinically protective, potentially leading to the minimization of inhibitor potency [21].

The development of AHA is closely linked to immune dysregulation, which leads to the production of neutralizing autoantibodies against factor VIII. This breakdown in immune tolerance is frequently observed in autoimmune diseases such as systemic lupus erythematosus (SLE), RA, and autoimmune thyroiditis [22]. These conditions involve aberrant immune activation, autoantibody production, and chronic inflammation, which may contribute to the emergence of AHA. Additionally, diseases such as pemphigus—a group of autoimmune blistering disorders—have also been reported in association with AHA [23]. Pemphigus is characterized by the loss of self-tolerance to epidermal adhesion proteins, and its coexistence with AHA further supports the theory that immune dysregulation plays a central role in the development of inhibitor formation. Apart from autoimmune diseases, AHA has been linked to malignancies, particularly solid tumors and lymphoproliferative disorders [14]. These malignancies often result in immune system perturbations that may contribute to the emergence of autoantibodies. Similarly, infections and certain medications, including penicillin and type-I interferons, have been implicated in triggering AHA, possibly by stimulating immune activation or molecular mimicry mechanisms [8]. While idiopathic cases of AHA have been reported, their true idiopathic nature is debatable. Given the extreme rarity of the disease (approximately two cases per million per year), the available literature suggests that many cases previously classified as idiopathic were later found to be associated with underlying autoimmune or neoplastic conditions upon further investigation [22]. This raises the question of whether truly idiopathic AHA exists or whether an underlying immunological trigger remains undetected in these patients.

Based on our findings and the available literature, we argue that AHA should be viewed primarily as a secondary phenomenon rather than an isolated disease entity. Its strong association with RA and autoimmune thyroiditis suggests that AHA is rarely, if ever, a standalone condition. Instead, it appears to be an immune-mediated complication of a broader autoimmune process [8,23]. Thus, in clinical practice, diagnosing AHA should prompt an in-depth search for an underlying autoimmune disorder or malignancy. This approach is essential for both understanding the pathogenesis of the disease and optimizing patient management.

**Figure 4:**
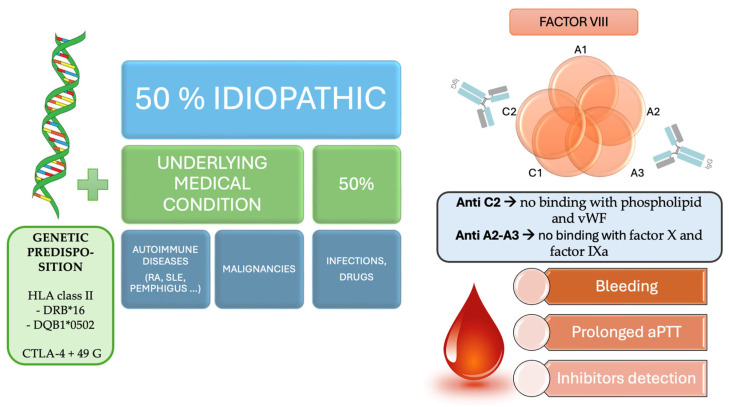
AHA pathogenesis. HLA: human leukocyte antigen; CTLA-4: cytotoxic T-lymphocyte associated protein 4; RA: rheumatoid arthritis; SLE: Systemic lupus erythematosus; vWF: von Willebrand factor; aPTT: activated partial thromboplastin time.

As an autoimmune disease, both genetic and environmental factors cause a tolerance breakdown with the production of IgG against FVIII. Among genetic predispositions, the association of human leukocyte antigen (HLA), class I and class II alleles, and single nucleotide polymorphisms (SNPs) of the cytotoxic T-lymphocyte associated protein 4 (CTLA-4) gene (318 C/T, +49 A/G, and CT60 A/G) with AHA have been investigated. Higher levels of the HLA class II DRB*16 and DQB1*0502 alleles and the CTLA-4 + 49 G allele were observed more frequently in patients with AHA [24]. The immune dysregulation observed in autoimmune conditions, associated in approximately 10% of AHA cases, may contribute to the development of inhibitory antibodies against FVIII. Similarly to other autoimmune diseases, peak incidence in older age plays role in AHA development [25]. Age-related changes such as alterations in T-cell function, impaired regulatory mechanisms, and amplified susceptibility to autoimmune responses due to cumulative antigen exposure throughout life may play a crucial role [26]. Moreover, dysbiosis, characterized by alterations in the composition and function of the microbiota with age, has also been implicated in enhancing autoimmune diseases among older adults [27]. AHA pathogenesis, among autoimmune disorders, is characterized by a complex relationship between aging and immune dysregulation. Figure 4 summarizes the pathogenesis of AHA.

RA and connective tissue disease, regardless of disease activity, are the main autoimmune diseases associated with AHA, as reported by several cohort studies [5,13]. An important point to emphasize is that the use of biologics should be carefully evaluated in patients with RA associated with AHA. Several cases of AHA have been reported during therapy with TNF inhibitors (TNFi). Specifically, one case of AHA in a 47-year-old woman with rheumatoid arthritis (RA) saw the patient develop spontaneous ecchymosis and hematoma eleven months after starting etanercept due to the presence of an anti-factor VIII antibody. Treatment included the discontinuation of etanercept and the administration of high-dose prednisone along with rituximab and azathioprine, which eventually led to remission [28]. In another case, a 66-year-old woman with RA developed acquired hemophilia during treatment with certolizumab pegol. Treatment with rituximab successfully controlled the condition [29]. In another report, a 68-year-old man with a history of RA and long-term immunomodulatory treatment developed severe spontaneous bleeding, including gastrointestinal bleeding, macroscopic hematuria, and extensive hematomas due to anti-factor VIII antibodies after treatment with adalimumab. The patient had to follow an immunosuppressive protocol including cyclophosphamide, dexamethasone, and rituximab [30]. Although the mechanism by which the TNF antagonist could potentially initiate the development of anti-factor VIII antibodies remains elusive, the efficacy of rituximab in refractory cases underscores the role of B cells in the pathogenesis of both RA and AHA [31,32].

Another interesting aspect is the evidence that joint damage in RA and arthropathy due to AHA cutting have some common pathogenetic factors. In RA, the inflammatory process is mediated by immune cells that release pro-inflammatory cytokines, including TNF-α, IL-6, IL-1β, IL-17, and IL-23, leading to the erosion of cartilage and bone [33]. In AHA, rare arthropathy results from recurrent intra-articular hemorrhages that cause the deposition of hemoglobin-derived iron in joint tissues. This event triggers an inflammatory response in the synovium like that of RA, with the release of cytokines such as IL-6 and TNF-α, which contribute to the progressive destruction of the joint [34]. Finally, angiogenesis has been reported to play a role in the pathogenesis of RA and AHA. Some evidence has shown that increased vascularization contributes to inflammation and joint destruction. Therefore, it has been hypothesized that targeting angiogenesis-related pathways may offer new therapeutic strategies to slow disease progression and reduce inflammation, including VEGF inhibitors, anti-TNF therapies, and modulators of angiogenesis, which could help manage symptoms and prevent long-term joint damage [35].

In recent years, some cases of AHA associated with IgG4-RD have been reported in adults and in children but the underlying mechanism remains unknown. It has been hypothesized that autoantibody production results from an oligoclonal proliferation of IgG4+ plasma cells driven by reduced tolerance to endogenous FVIII. It is also possible that the anti-FVIII activity could be the result of autoantibody production by uninvolved cells due to polyclonal IgG4+ plasma cell proliferation [36,37]. Another association of AHA is with bullous pemphigoid, a common autoimmune subepidermal blistering disease characterized by the production of autoantibodies against the hemi-desmosome proteins BP antigen 230 (BPAg1) and BP antigen 180 (BPAg2). Also, in this case the association between these autoimmune disorders remains unknown but a possible explanation is provided by cross-reacting antibodies, in which the IgG1 and IgG4 subclasses in bullous pemphigoid bind the A2 domain of factor VIII [38].

Rarely, isolated thyroid involvement is associated with the onset of AHA [39]. In the literature, different case reports describe associations between Hashimoto thyroiditis and AHA, usually in combination with another autoimmune disorder, for example, pangastritis or pemphigus [40,41].

AHA deserves specific mention after vaccination against coronavirus disease 2019 (COVID-19). As of 2021, several autoimmune diseases have been reported to have risen after the administration of mRNA vaccines against COVID-19; immune thrombocytopenic purpura (ITP) has been the most frequently described among coagulation disorders, but AHA has also been observed, even with spontaneous resolution. It is probably random, but several cases of this type have already been reported [42]. One possible explanation is that vaccines can promote or enhance immune reactions, followed by the onset of new autoimmune events or the exacerbation of pre-existing but latent immune dysregulations, which can consequently drive the clonal selection of B cells that produce specific autoantibodies, including those against FVIII [43]. Furthermore, some cases of AHA following flu vaccinations have been reported [44].

In 2009, Huth-Kühne et al. published international recommendations on the diagnosis and treatment of AHA, and subsequently, other guidelines were also published, based on the collective experience of authors in treating many patients with AHA [1]. Since then, data from several AHA registries have been published, including the EACH2 (European Acquired Haemophilia) [4,45], SACHA (Surveillance des AutoantiCorps au cours de l’Hémophilie Acquise) [46] and GTH (Gesellschaft für Thrombose- und Hämostaseforschung) registries [47] in Europe, as well as the HTRS (Hemostasis and Thrombosis Research Society) registry in the United States [48]. In 2020, an updated set of recommendations was published, based on a higher level of recent evidence [8]. Usually, diagnosis is made in an emergency or internal medicine department and then the treatment is followed up by hematologists. Typically, AHA diagnosis is suspected when patients present acute or recent bleeding symptoms, without a previous history of bleeding, with laboratory data presenting an isolated prolonged APTT, reduced FVIII activity (<1% in 50% of cases; <5% in 75% of cases; <40% in 100% of cases), and the presence of autoantibodies, detected by the Bethesda assay. If the prothrombin time (PT) is prolonged, it must be attributed to other causes, e.g., anticoagulant treatment. AHA should be suspected in a nonbleeding patient not on anticoagulation medicine with an isolated prolonged aPTT, a mixing study consistent with an inhibitor, and a negative LA.

The management of AHA should achieve the following objectives: the reduction and prevention of bleeding, inhibitor eradication, and the management of the underlying disease. For the first objective, replacement with human FVIII is not effective in the presence of high titer inhibitors [49]. Recombinant factor VIIa (rFVIIa, NovoSeven^®^) and activated prothrombin complex concentrate (aPCC, FEIBA^®^—plasma-derived, containing factors II, IX, and X and VIIa) are both appropriate first-line bypassing treatments. While the efficacy of rFVIIa in AHA was recently addressed in a systematic literature review, no systematic reviews are available for APCC, which is used for the treatment and prevention of bleeding in patients with congenital hemophilia with inhibitors and is widely used for AHA [50,51,52]. Both agents control about 80% of bleeding episodes in patients with hemophilia and inhibitors, with a favorable safety profile indicating that they are well-tolerated with few adverse events. Thrombotic events have been reported mainly in patients with additional risk factors and disseminated intravascular coagulation has been observed after high-dose APCC administration [53]. If first-line drugs are not available, second-line of treatment can be used as well as recombinant porcine factor VIII, human factor VIII concentrates, and desmopressin [54]. Immunosuppressive treatment is equally important, and should be started immediately after diagnosis, to reduce the risk of bleeding and to try to achieve AHA remission as quickly as possible. Complete remission is obtained when FVIII is normal, inhibitors are undetectable, and immunosuppression has stopped or been reduced to doses used before AHA developed without relapse. Partial remission has also been defined as a recovery of FVIII > 50% and the absence of bleeding, after the discontinuation of any hemostatic treatment for at least 24 h [5]. Initial treatment with corticosteroids (1 mg/kg/day) alone or in combination with cyclophosphamide for up to 6 weeks is usually recommended, while second-line therapy with rituximab is suggested if first-line treatment failed or was contraindicated. Rituximab should be used as a first-line treatment in patients with poor prognostic markers (FVIII < 1 IU/dL or inhibitor titer > 20 BU) [8]. Old age, with its comorbidities and medications, such as antiplatelet agents and anticoagulants, as well as thrombocytopenia associated with autoimmune diseases can worsen the clinical picture and make the management and treatment of AHA more delicate and difficult [55]. Patients’ follow-up must be very close until complete remission and for several months thereafter, as adverse events (occurring in 66% of patients) have also occurred after more than 100 days (25%). The monitoring of FVIII activity is more sensitive than APTT for detecting relapses.

## 4. Conclusions

AHA is a rare but life-threatening condition with a significant mortality rate. Prompt diagnosis is crucial, as is early therapy, for hemostasis and inhibitor eradication can be lifesaving, improving AHA outcomes. Special attention is required for patients with autoimmune conditions or malignancies, elderly patients, and patients treated with antiaggregant and anticoagulant agents; although rare, AHA must be suspected, especially in these high-risk populations. Our case report suggests that rheumatoid arthritis should be rarely associated with AHA. Better knowledge and recognition on the part of different specialists could ensure that AHA patients receive the timely and personalized treatments they need.

## Figures and Tables

**Table 1:** Laboratory data.

Variable	In Medicine Department	Reference Range
Creatinine (mg/dL)	0.62	0.50–0.9
Na^+^ (mmol/L)	140	136–145
K^+^ (mmol/L)	4.5	3.4–5.5
Ca^+^ (mmol/L)	2.11	2.10–2.50
Proteins (g/L)	59	60–82
Albumin (g/L)	38	35–55
Amylase (U/L)	68	28–100
Lipase (U/L)	40	13–60
AST (U/L)	8	9–45
ALT (U/L)	14	10–40
Total Bilirubin (mg/dL)	1.45	0.30–1.20
Direct Bilirubine (mg/dL)	0.51	<0.20
CPK (U/L)	693	20–200
LDH (U/L)	365	135–225
Haptoglobin (mg/L)	240	300–2000
Folate (ng/mL)	>20	3.8–16.0
Vitamin B12 (pg/mL)	215	191–663
Ferritin (µg/L)	73	30–400
Reticulocytes (%)	5.99	0.50–2.50
CRP (µg/L)	22,800	100–6000
ESR (mm/h)	47	0–35
Hb (g/dL)	8.2	12.2–15.3
WBC (×10^9^/L)	10.72	4.40–11.30
N (×10^9^/L)	7.39	1.80–7.70
L (×10^9^/L)	2.18	1.80–4.80
PLT (×10^9^/L)	469	150–450
Fibrinogen (g/L)	5.50	1.50–4.00
INR	0.92	0.81–1.20
aPTT ratio	1.91	0.8–1.30
C3 (mg/dL)	136.00	90.00–180.00
C4 (mg/dL)	32.30	10.00–40.00

Na^+^: sodium; K^+^: potassium; Ca^+^: calcium; AST: aspartate aminotransferase; ALT: alainine aminotransferase; CPK: creatine phosphokinase; LDH: lactate dehydrogenase; CRP: c-reactive protein; ESR: erythrocyte sedimentation rate; Hb: hemoglobin; WBC: white blood cells; N: neutrophils; L: lymphocytes; PLT: platelets; INR: international normalized ratio; aPTT: ratio activated partial thromboplastin time ratio; C3/4: complement 3/4.

## Data Availability

Data are contained within the article.
